# Socrates’s Last Words to the Physician God Asklepios: An Ancient Call for a Healing Ethos in Civic Life

**DOI:** 10.7759/cureus.3789

**Published:** 2018-12-28

**Authors:** James E Bailey

**Affiliations:** 1 Internal Medicine, University of Tennessee Health Science Center, Memphis, USA

**Keywords:** asklepios, ascelepius, ethics, plato, socrates, history of medicine, public health, medical education, palliative care, professionalism

## Abstract

Socrates’s last words have remained enigmatic despite over two millennia of philosophical, literary, and historical interpretations. This paper suggests that Socrates was executed for questioning the imperialistic actions of Athens in the Peloponnesian War by elevating the emerging cult of Asklepios and advocating for Asklepian ideals. Plato’s dialogues show that Socrates saw Asklepios as more worthy of emulation than the warlike gods of the state-supported Greek pantheon. While dying from the executioner’s hemlock, Socrates asks his friend Crito to pay the traditional thank offering given to the physician-god: a cock symbolizing rebirth. He looks to the only god then known to revive the dead to help his ideas and spirit live on. Socrates’s last words thwart Athenian authorities’ attempts to silence him, issue a call for Asklepian ideals to prevail in the city of Athens, and identify the selfless caring for others exemplified by Asklepios as the highest duty for all humans. Socrates calls us from the past to remember timeless Asklepian physician duties to self, patients, and community. Socrates reminds modern physicians of their personal duty to make their own spiritual health their first priority, their professional duty to comfort the sick and alleviate suffering, and their societal duty to advocate for the vulnerable, sick, and suffering and the health of the public.

## Introduction and background

Crito, we owe a cock to Asklepios - Pay it and do not neglect it [1].

Socrates’s dying words to his students call for an offering to Asklepios, the physician hero and demi-god newly introduced to Athens during the time of Socrates. Multiple literary sources confirm that Socrates was prosecuted for not believing in the state’s gods, introducing new gods, and corrupting the youth with these ideas. Why does one of the Western world’s greatest and most original philosophers use his last words to give thanks to a healing god? Was Socrates executed by hemlock in part because of his pious support for Asklepios’ emerging healing cult? 

This article explores the meaning of Socrates’s dying words to his students and their relevance for modern physician-teachers and our students. The article seeks to discover both what Socrates’s death can tell us about physician-healer duties to self, patients, and society. We will discover that Socrates’s Asklepian ideals provide guidance to modern physicians on how to comfort, alleviate suffering, and assist in promoting life in the face of serious illness. These ideals also call physicians to fulfill fundamental obligations to stand up for the weak, vulnerable, sick, and suffering and to work on behalf of public health.

## Review

To understand Socrates’s last words and the reasons for his execution and death, we must first learn a little of what Greeks in ancient times knew about Asklepios. The earliest references to Asklepios, found in Mycenean inscriptions, date to approximately 1500 BC. Homer’s *Iliad* (circa 900 BC) mentions Asklepios’ sons and characterizes Asklepios as “a gentle craftsman” who cares deeply for all humans. Homer portrayed Asklepios as a mortal physician hero, not a god. However, Asklepios was the only Greek hero known for feats of healing. He dared to raise from death even heinous criminals condemned by the gods, and Zeus executed him for this transgression [2-3].

Pindar, the famous Greek poet, suggests that Asklepios raised someone from the dead for money [3}. In *The Republic*, Socrates disagrees with Pindar’s conception of Asklepios, saying, “if he was a god's son, we'll say he wasn't basely greedy, and if he was basely greedy, he wasn't a god's son” [4]. The more traditional Homeric gods, on the other hand, were always viewed as capricious. Xenophanes attributed to the gods “all of the acts which are counted by men disgraceful and shameful [5]. Solon stated, “Everything divine is envious and meddlesome” [6]. However, the overwhelming majority of depictions of Asklepios concur with Socrates’s view that Asklepios, unlike the gods of the traditional Greek pantheon, was “blameless” [2,5].

In Socrates’s time, Asklepios was gaining recognition as a new physician-god. Supplicants sought healing at Asklepeions, the temples to Asklepios, all over Greece and Asia Minor [7] (Table 1).

**Table 1:** Key events in the time of Socrates

Year (BC)	Events
500	First altar and sacred building for Asklepios erected in Epidauros [2]
475	Pindar composes a poem to Asklepios speaking of him as a hero, not a god.
464	Socrates’s birth in Athens
431	Peloponnesian war starts [8], and Oracle at Delphi predicts Athens's loss.
430	Pericles, the leader of Athens, attacks Epidauros in the first phase of the Peloponnesian war. Plague strikes Athens, and Athens erects a statue to Apollo in thanks for bringing an end to the plague in that year [8].
429	The plague returns. Pericles dies of unknown causes [9].
427-426	The plague returns a third time. When it finally ends, the state credits Apollo with saving Athens from the epidemic [10-11].
425?	Delphic Oracle answers that Socrates is the wisest man in Athens; Socrates interprets that he is wisest only in that he knows that he knows little [12].
423	Aristophanes’ comedy Clouds makes fun of Socrates’ conception of the gods.
421	Free association between Athens and Epidauros restored with Peace of Nikias [8]
420	Telemachus, a private individual, brings worship of Asklepios to Athens, founding a private shrine dedicated to his worship on the south slope of the Acropolis [13-14]. Government approves private construction of the temple as it does for most foreign gods. Asklepios accepted in Athens [9,15-16]. Athens again on brink of war with Epidauros [8].
411	Sparta supports the coup in Athens. An oligarchic government of 400 aristocrats is installed but is overthrown in four months [8,17].
410	Asklepios honored with a sacred precinct in Delphi [18].
408	Aristophanes’s Plutos first performed making fun of priests and healing rituals in the Asklepeion temples.
404	Peloponnesian War ends when Athens surrenders to Sparta. Sparta installs 30 tyrants over Athens [8,17,19].
403	Thirty tyrants overthrown, democracy restored [8,19-20].
401	Oligarchs likely planned a third coup attempt [17].
399	In the Euthyphro, Socrates goes to answer charges of impiety. In the Apology, Socrates is tried before a court of 500 Athenian citizens, is convicted by a small margin, and sentenced to death. In the Crito, Socrates's best friend named Crito tries to convince Socrates to escape and flee Athens, but Socrates argues that it would not be honorable. In the Phaedo, Socrates is executed 30 days after his trial by being required to drink a potion of hemlock. Socrates's friend Xenophon is exiled to Sparta [17].
360- 340	Cult of Asklepios is officially recognized by the state, comes under state control and is overseen by the democracy’s Council of 500 [13,20].

But the worship of Asklepios was newly introduced in Athens during Socrates’s middle age. The Asklepeion in Athens was built with private funds without formal state sanction far from the city center. Yet the ideals promoted at the Athenian Asklepeion are clear and different from those of the state-supported gods in Athens. The temple bears this inscription: “These are the duties of a physician...he would be like God savior equally of slaves, of paupers, of rich men, of princes, and to all a brother, such help he would give” [2-3,21].

Evidence from the Platonic dialogues

Much of what we know of Socrates and his ideas comes from the dialogues, authored by the Athenian aristocrat Plato. These works are based on the first- and second-hand accounts of Socrates’s conversations with students and adversaries in Athens.

Most authors deem these accounts generally reliable since they were published when many of the discussants could attest or dispute their accuracy [22-25]. The 500 Athenian citizens who witnessed Socrates’s defense would have been outraged if they had seen major inaccuracies in Plato’s *Apology*. Additional contemporary historical accounts corroborate the dialogues’ depiction of the charges, trial, conviction, and execution of Socrates [26-28]. 

Throughout the dialogues, Socrates challenges the traditional Homeric conception of the gods that the state adopted as war propaganda. Athens was an aggressive imperialist state during this time, charging protection fees to all the states in the Delian league, and starting the Peloponnesian War to protect its foreign interests. In the *Phaedo *Socrates notes, “for all wars arise for the sake of gaining money...” [29]. When he challenged the use of the state-approved gods to justify the pursuit of wealth and power, he publicly questioned the purposes of the state and its war.

Socrates’s conviction for introducing new gods

**Figure 1:**
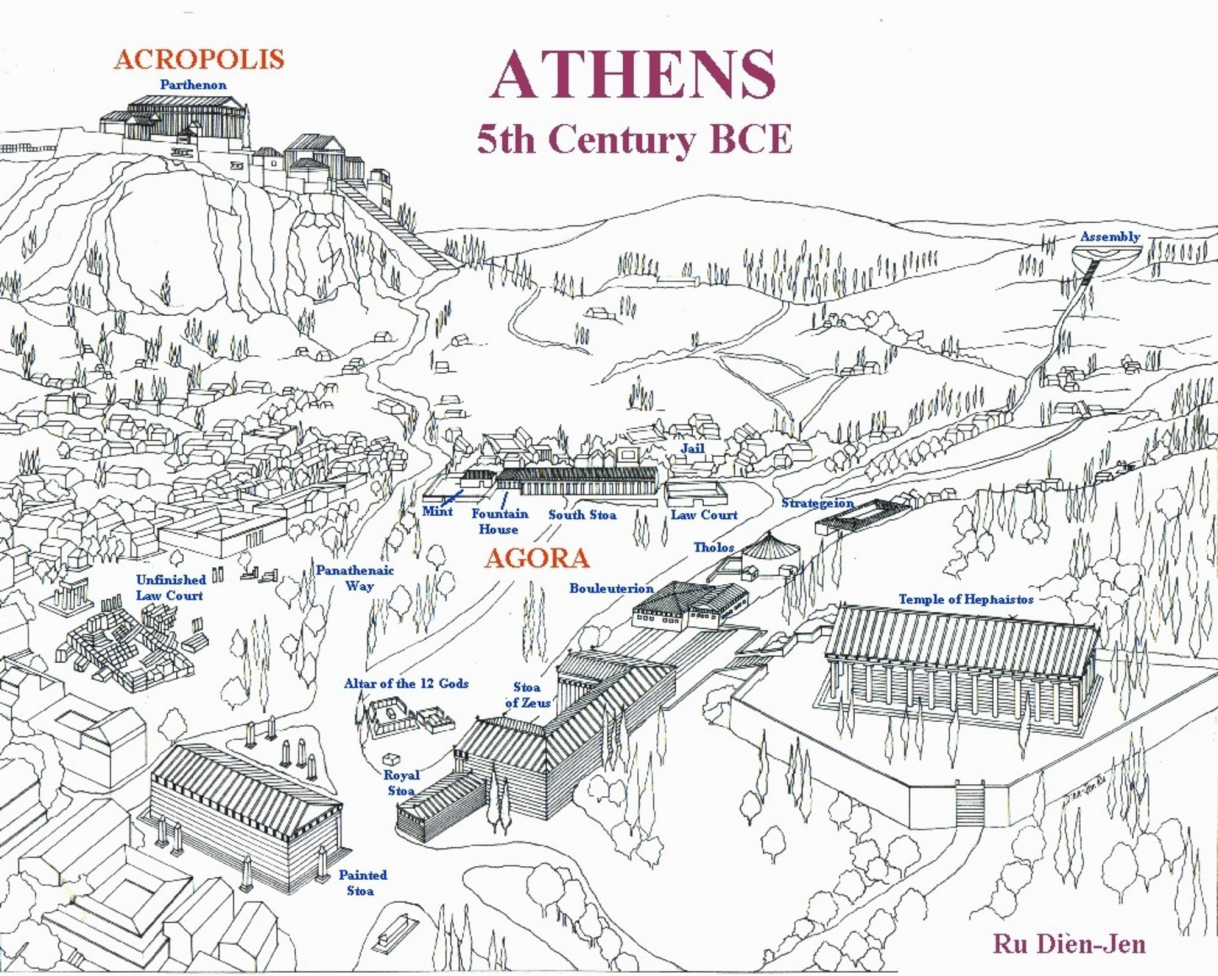
Athens as it appeared in the 5th century BC The Altar of the 12 gods and the Stoa of Zeus, where Socrates was charged, are shown in the foreground of the Agora in the center of Athens. The Assembly is shown in the upper right where Socrates was tried, convicted and gave his apology. In the background of the Agora is the jail where he was held for 30 days and executed. The Asklepeion of Athens is far out of the center of Athens on the backside of the Acropolis and is not seen in this view. Drawing by Ru Dien-Jen from http://socrates.clarke.edu/athens.gif. Used by permission.

In the *Euthyphro*, Socrates goes to a porch of the Temple to Zeus, facing the Altar of the Twelve Gods to answer the charge of introducing new gods (Figure 1) [23]. This altar in the center of the agora was dedicated to the twelve major gods of Athens: Zeus, Hera, Poseidon, Demeter, Hestia, Apollo, Artemis, Hephæstus, Athena, Ares, Aphrodite, and Hermes [30].

Asklepios was not represented in the agora. His new temple was far from the city center of political power. Socrates could have looked to Apollo as the representative healing god whom the state credited with averting the plague (Table 1). Instead, Socrates recognizes Apollo’s son, Asklepios who was delivered from his dying mother’s womb by his father in the first Apollonian delivery [2-3,10]. At this official place of public worship to the twelve major gods, Socrates challenges the very character of the traditional gods by arguing that real gods could never be biased or deceitful [23].

The Oracle of Delphi had a significant relationship to Asklepios, Apollo, Athens, and Socrates during this period. The Pythia, Apollo’s priestesses at Delphi, were named for the Python, a great dragon snake killed by Apollo at Delphi, the center of the world. The highest priestess of the Pythian Apollo served as the Oracle, communicating with the spirits of the underworld and relating truths from the spiritual realm. Asklepios was a chthonic deity, a god in touch with the earthly underworld, the world of spirits, like Demeter, Pluto, and Orpheus who were worshiped at Delphi [2,10,31]. Like many other chthonic deities, Asklepios was represented as a snake or with a snake, the ancient healing symbol still used to represent the power of medicine.

Although Athens controlled Delphi politically, the messages of the Pythia favored Sparta during the Peloponnesian War, causing resentment in Athens. Simultaneously, Delphic oracles aggressively promoted Asklepios and favored Epidauros, Sparta’s neighbor, as the center of Asklepian worship (Table 1). During this period, Athenians regarded Delphi, Epidauros, and Asklepios with suspicion, but Socrates had great respect for the Delphic oracle. Likewise, the Delphic oracle had great respect for Socrates, naming him the wisest of all Athenians [12]. 

At trial, Socrates was accused of rejecting the state’s gods (Greek: *theous*), introducing new spiritual beings (Greek: *daimonia kaina*) and corrupting the youth with these ideas. Garland notes it is Socrates’s new way of viewing the gods that Aristophanes satirizes in his play “The Clouds” that was well known to all Athenians [32, 33]. Some have suggested that the only new god Socrates was convicted of promoting was his own inner voice, which he likened to a personal spirit (Greek: *daimonion*) that spoke to him in the same way the god spoke directly to the Pythia at Delphi, a bold assertion that further threatened the authority of the Athenian government [22,27,34].

Socrates was likely viewed as a pro-Spartan because of his perceived support for oligarchy and rule by the best, rather than full participatory democracy [35]. He educated the same type of aristocratic youth that connived with Sparta to overthrow the democracy in 411, 404, and 401 BC (Table 1) [17]. Socrates denied participating in these plots, and substantial evidence indicates his strong preference for democracy. However, it remains likely that the Athenians feared he would corrupt youth with Spartan political ideas [17,35-36].

Socrates’s dying words of thanks for healing

In the *Phaedo*, the dialogue depicting Socrates’s final conversation and execution, Plato focuses on what the transition of death means for the spiritual health of the individual. Socrates identifies the mind or soul (Greek: *psyche*) as the natural leader of the individual, one’s essential and immortal part. The body, which racks a person with pleasures and pains, imprisons the soul until it finds freedom and healing in death. 

Socrates says the soul of the philosopher seeks to free itself from the bondage of bodily pleasures and pain, to abide with the divine “making that its only food… to be free from human ills” [37]. The individual virtues first introduced in *The Republic* – self-restraint, justice, courage, and wisdom – “are a kind of purification” [37] from the pleasures, pains, and concerns of the body.

Socrates notes that the soul upon death “goes away into that which is like itself, into the invisible, divine, immortal, and wise, and when it arrives there, it is happy to be freed from error and folly and dear and fierce loves...” [37]. Once free from the body and its impurities, one can finally attain the truth. Socrates encourages his friends by telling them of the heavenly world of mind, ideas, and soul, describing it as an “upper world” of ideal forms, “pure, not corroded or defiled, as ours is, with filth and brine … which causes ugliness and diseases” [37].

When two of the students, Simmias and Cebes, express their persistent fear of death, the group recognizes Socrates as their physician. The narrator Phaedo notes “the skill with which he cured us” [37]. Socrates says they have no reason to mourn unless their fears of death overcome their souls, and he sets out as their physician to cure them of these fears.

Although Socrates begins his discussion with his friends in the *Phaedo* by recognizing death as a great good, he acknowledges that people, as proper servants to the gods, have no right to take their own lives, but “must wait for some other benefactor” [37]. Contrary to the popular belief that Socrates committed suicide, most commentators agree that he did not [9,17,33,38]. Rather, he was executed by a city-state that was threatened and angered by his criticism. It is true that Socrates could have avoided execution by fleeing his beloved homeland and living in exile for the rest of his life. In fact, Socrates’s friends encouraged him to escape and avoid execution in the *Crito* and *Phaedo*. But Socrates refused this easy path out because of his respect for Athens’ democratic constitution [35], and thus he was duly executed by the very people and the government he honored.

The hemlock pharmakos

Socrates’s last words are not incidental, but rather are carefully staged by Plato. In the last minutes of his life, Socrates is given two initial opportunities to charge his followers with special duties following his death. First, Crito asks him specifically if he has any special requests for his friends. Then, when Socrates receives the potion of hemlock, he asks if he can pour out a portion of it as a libation to the gods. When he is told that the full draught is necessary to give him a sufficient dose, he instead offers a prayer to the gods for a safe passage. Only at the very brink of this passage—as the sun sets, and after he has taken the poison, laid down, covered his face, and felt his body begin to go numb—does he uncover his face one last time and ask that a debt be paid to Asklepios. The deliberate timing of Socrates’s final request suggests that Socrates recognizes that healing has begun to occur. How could this be?

**Figure 2:**
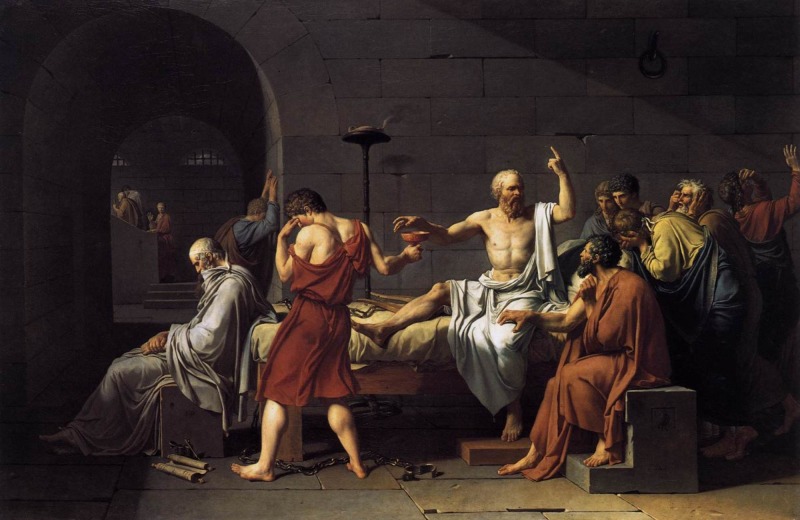
Socrates depicted as both doctor and patient in his last hour Jacques-Louis David, The Death of Socrates, 1787. Metropolitan Museum of Art, New York. Public Domain.

The Greek word *pharmakos* means both poison and remedy [7]. Socrates sees the hemlock as a medicine that frees him from the fetters of pain and pleasure (Figure 2). The oldest meaning of the word *pharmakos* is scapegoat, the blameless victim that was sacrificed to take the blame merited by the unhealthy citizens and to carry disease and pestilence from the town. This meaning is specifically evoked by the imagery that surrounds the conviction and death of Socrates.

The *Phaedo* opens with a description of the annual commemorative voyage conducted in thanks to the god Apollo for his saving of the best of the youth of Athens from certain death. On the day of Socrates’s conviction, a ship leaves for Crete commemorating the journey of Theseus and 13 other youth and maiden *pharmakoi* sent to be sacrificed to the Minotaur. In this mythical journey, Theseus took the place of the 13 others and killed the Minotaur in the depths of the labyrinth. By law, all executions were deferred until the return of the commemorative ship, and Socrates’s execution in the *Phaedo* occurs on the day of its return (Table). Socrates is depicted as a new Theseus who takes the place of his 13 friends present at his execution [38]. The *Phaedo* ends with a thanks offering to Apollo’s son Asklepios for a different sort of rescue, the rebirth of the soul after death.

Socrates’s taking of the pharmaceutical hemlock evokes meaning at two major levels. First, the potion cures him personally of the fever of life, purifying him and allowing him to enter the spirit world. Second, the potion reminds us that Socrates himself serves as a *pharmakos* for his young friends. He both takes the blame that they might merit as young aristocrats fomenting change in Athens and cures them of their fear of death. He purges them of their love of wealth, power, honor, and the physical body and fosters their love of knowledge and the universal ideas of the spiritual world. Throughout the dialogue, he recognizes the dual nature of *pharmakoi*; all such cures are also potential poisons, and life comes from death.

The jailer, who carries out Socrates’s execution by giving him hemlock, transforms from executioner into pharmacist-healer. As the potion causes Socrates’s body to chill, he loses feeling and awareness of his body. The numbness slowly moves up from Socrates’s feet to his thighs. The man administering the poison pinches his feet hard, and Socrates does not feel it. When the chill reaches his groin, Socrates gives us his last words: “Crito, we owe a cock to Asklepios - Pay it and do not neglect it” [1].

Why did Socrates choose these words about Asklepios as his last? Although many previous authors have speculated about this, [9,17,27,32-35,37,39], they have not fully recognized the importance of Asklepian ideals in Socratic thought. Socrates was clearly guilty of rejecting the warlike gods promoted by the state, advocating the worship of new and better gods like Asklepios, and educating the youth with these ideas.

Asklepios was known as the “good physician,” who so loved others that he lost his own life bringing others back from the dead [3,40]. The cock, which gives hopeful proclamation of the coming new day, symbolized rebirth and afterlife for ancient Greeks and was the traditional thank offering given to the healing god Asklepios [8]. Socrates is simply offering thanks and pointing to the afterlife. He invokes the only god known to revive the dead, who Socrates suggests with his last words has already helped heal both Socrates himself and his followers from the fever of earthly life [41-45].

Socrates calls us from the past to honor timeless Asklepian ideals for the benefit of ourselves, our patients, and our community. In his last words, Socrates remembers his student-friends, saying that “we” owe a debt to the healing divinity [1]. By remembering others in his last moments, Socrates exemplifies the healer's virtue of altruism and gives guidance to medical educators today regarding the level of devotion the best teachers have for their students and how they strive to heal their students' ills.

On a personal level, Socrates reminds his followers to make their own spiritual health their first priority. His words evoke the ancient Greek proverb, “Physician, heal thyself”. Socrates calls physicians to life in recognition that they, along with all their patients, are on a path to healing. He thanks Asklepios for his release from earthly concerns and passage to a new life in the spiritual world, and for his students’ release from their fear of death. On a professional level, Socrates’s words encourage modern physicians to emphasize patient comfort, the alleviation of suffering, and the promotion of abundant life even at the time of death. His words suggest that physician healers, like Asklepios, have a moral obligation to speak out for the weak, vulnerable, sick, and suffering. On a societal level, Socrates uses his last words to thwart the Athenian authorities’ attempt to silence him. Like Asklepios, he is executed for his commitment to healing. He willingly serves as a scapegoat for his friends and as a martyred social advocate for his beloved city. Socrates’s Asklepian ideals call modern physicians to speak out on issues such as gun policy where private interests may conflict with the best interest and health of the public.

## Conclusions

Socrates’s final words, long remembered and often repeated, call for Asklepian ideals to prevail in the city of Athens. Evidence from first-hand accounts of Socrates’s conversations with his students, trial, conviction, and execution suggests that Asklepios was among the new gods about whom Socrates was accused of educating the youth. Thus, Socrates was likely executed in part because of his pious support for Asklepios’s emerging healing cult in Athens. The Asklepian healing ideals for which Socrates gave his life still hold relevance for physicians today. Socrates calls us from the past to remember timeless Asklepian physician duties to self, patients, and community. Socrates reminds modern physicians of their personal duty to make their own spiritual health their first priority, their professional duty to comfort the sick and alleviate suffering, and their societal duty to advocate for the vulnerable, sick, and suffering and the health of the public. Socrates admonishes his friends and fellow citizens to work to purify their souls, to serve others with compassion, to reject petty gods and selfish imperialistic war, and to dedicate their lives to the community’s health. Socrates’s last words remind healers and patients alike to participate in and give thanks for the wondrous gift of true healing.
